# Acute appendicitis in preschoolers: a study of two different populations of children

**DOI:** 10.1186/1824-7288-37-35

**Published:** 2011-07-25

**Authors:** Stefanos Gardikis, Alexandra Giatromanolaki, Katerina Kambouri, Gregorios Tripsianis, Efthimios Sivridis, George Vaos

**Affiliations:** 1 Department of Pediatric Surgery, Alexandoupolis University Hospital, Democritus University of Thrace School of Medicine, Alexandroupolis 68100, Greece; 2 Department of Pathology, Alexandoupolis University Hospital, Democritus University of Thrace School of Medicine, Alexandroupolis 68100, Greece; 3 Department Medical Statistics Alexandoupolis University Hospital, Democritus University of Thrace School of Medicine, Alexandroupolis 68100, Greece; 4 Laboratory of Experimental Surgery and Surgical Research, Alexandoupolis University Hospital, Democritus University of Thrace School of Medicine, Alexandroupolis 68100, Greece

**Keywords:** Abdominal pain, Acute appendicitis, Household amenities, Child, Preschool ages, Lymphoid tissue

## Abstract

**Objective:**

To assess the incidence and the risk factors implicated in acute appendicitis in preschoolers in our region.

**Methods:**

Over a 7-year period, 352 children underwent appendectomy for suspected acute appendicitis. Of these, data for 23 children were excluded because no inflammation of the appendix was found on subsequent histology. Of the remaining 329, 82 were ≤ 5 years old (i.e., preschool children) and 247 were 5-14 years old. These two groups of children were further divided according to their religion into Muslims and Christian Orthodox: 43 of the children aged ≤ 5 years were Muslims and 39 were Christian Orthodox. A household questionnaire was designed to collect data concerning age, gender, type of residence area, living conditions, vegetable consumption, and family history of surgery for acute appendicitis as preschool children. The removed appendices were also assessed histologically for the amount of lymphoid tissue.

**Results:**

Acute appendicitis of preschoolers developed more frequently in Muslims (39.4%) than in Christians (17.7%; *p *< 0.001). The lack of inside toilet facilities at home, overcrowded living conditions, living in rural areas, and the amount of appendix lymphoid tissue were significantly more frequent among the Muslim preschool children (*p *< 0.05), while there were no statistically significant differences between Muslim and Christian children with regard to gender, the family history of acute appendicitis, or the vegetable consumption (*p *> 0.05).

**Conclusions:**

In our region, the percentage of preschool-aged Muslim children with acute appendicitis was remarkably high. One possible explanation for this finding could be the higher amount of lymphoid tissue in the wall of the appendix in Muslim preschool children together with their low standard of hygiene.

## Background

Acute appendicitis is the most common cause of acute abdomen requiring surgical intervention during childhood, accounting for 1-8% of children who present to the pediatric emergency room with acute abdominal pain [1]. Epidemiological data indicate that the incidence of acute appendicitis varies widely between countries, among regions within the same country, and between different racial and occupational groups [2,3]. Several investigators have documented a higher incidence of appendicitis among adolescents and young adults, whereas it is uncommon in preschool-aged children (i.e., ≤ 5 years old) [1,4].

While surgical treatment of acute appendicitis is well established, the etiology and pathogenesis of this condition remains unclear, but appears to be multifactorial. Obstruction of the appendiceal lumen by a fecalith and hyperplasia of the lymphoid follicle in the appendiceal wall have been proposed as common causes of acute appendicitis [5]. Primary bacterial and viral infections [6], blunt abdominal trauma, and ischemia of the appendix [7] have been also investigated as possible causes. It has also been suggested that there is a genetic predisposition [8] or a type I hypersensitivity reaction involved in the development of acute appendicitis [9]. Furthermore, two hypotheses have been proposed in this regard: the "hygiene hypothesis" [10] and the "diet hypothesis" [11].

According to the 2001 census, the population of west Thrace (in northern Greece) was 363,548 and comprised two religious populations: Christian Orthodox and Muslim. Children represented 15.8% of the total population (57,614), of which 41.6% (24,004 children) were Greek Muslims. This Muslim population comprised Pomaks and Roma, who generally have a low parental occupational status and low family income. Taking into account our clinical observation of an increased frequency of acute appendicitis in Muslim children (MC) aged ≤ 5 years (i.e., preschool age) in comparison to preschool Christian Orthodox children (COC) with the same diagnosis, a retrospective study was conducted to determine the relationship between the risk factors implicated in acute appendicitis and the development of acute appendicitis in two socioeconomically and racially different populations of preschool children in Thrace, northern Greece.

## Methods

During a 7-year period (January 2003-December 2009), a total of 352 children underwent appendectomy following a preoperative diagnosis of acute appendicitis in the pediatric surgery department at Alexandroupolis University Hospital. Written informed consent to participate was obtained from all parents, and this study was approved by the regional ethical committee. Clinical/operative data were reviewed for all children who required an operation for acute appendicitis. The patients were allocated to one of two age groups: preschool (≤ 5 years) and school age (5-14 years). These two age groups were further divided according to their or their parents' reported religious affiliation into Greek Christian Orthodox and Greek Muslim.

A questionnaire was designed to collect the following data from families with children aged ≤ 5 years old who had undergone surgery for acute appendicitis: age, gender, whether the family lived in an urban or rural area, living conditions (type of toilet facilities, presence of a hot water system, and overcrowding), frequency of vegetable consumption, and family history of acute appendicitis in first-degree relatives. Families were considered as living in overcrowded conditions when more than four persons lived in a house with only one or two rooms. The toilet facilities included the existence or absence of a washbasin and the toilet being located outside or inside the house. The frequency of vegetable consumption was categorized as either almost every day (i.e., five times or more per week) or infrequent (twice or less per week). The information was obtained by questionnaire either in a face-to-face interview with the parents or by telephone. The information from the questionnaire and medical records from each patient were grouped and validated.

All removed appendices from children aged ≤ 5 years were evaluated in order to assess the amount of lymphoid tissue in the appendiceal wall. The amount of lymphoid tissue in the appendiceal wall was also assessed in 40 preschool children (20 MC and 20 COC) who underwent an incidental appendectomy during laparotomy for other surgical pathologies (e.g., intussusception, torsion of the ovary, and Meckel's diverticulum) or an appendectomy for suspected acute appendicitis but with no inflammatory findings on histology. The appendiceal reaction was scored in a semiquantitative manner and the amount of the lymphoid tissue was recorded as low/unremarkable (+) or high/remarkable (++). This evaluation was based on at least four sections from each specimen, which were viewed at a magnification of × 200.

We decided to evaluate and compare the aforementioned factors relative to religious affiliation for two reasons: (i) because they have been cited in various sources as potentially causal risk factors related to acute appendicitis [12] and (ii) because religion itself may influence customs and lifestyle.

Statistical analysis of the data was performed using the Statistical Package for the Social Sciences (SPSS), version 14.0 (SPSS, Chicago, IL, USA). The Kolmogorov-Smirnov test for normality was performed. Normally distributed continuous variables are expressed as mean ± standard deviation values, while nonnormally distributed variables are expressed as median and range values. Categorical variables are expressed as frequencies (and percentages). The chi-square test was used to evaluate any potential association between two categorical variables. Discriminant forward stepwise analysis was used to characterize patients according to their religion. Multivariate stepwise logistic regression analysis was used to determine independent risk factors for low or high amounts of lymphoid tissue. The odds ratio (OR) was estimated to quantify the associations of the children's characteristics with (i) low or high amounts of lymphoid tissue and (ii) the complication rate. The mean duration of symptoms was compared between groups using the Mann-Whitney *U*-test. The incidence of appendicitis was estimated based on the census of 2001, and compared between groups using the OpenEpi program. All tests were two tailed and the level of statistical significance was defined as *p *< 0.05.

## Results

Of the 352 children who had undergone appendectomy, 23 (8 MC and 15 COC; 6 and 7 of them, respectively, aged ≤ 5 years) were excluded from further analysis because the histology of the appendix was negative for acute inflammation. Of the remaining 329 children, who were aged 17-167 months (age 89.25 ± 38.91 months), 82 (24.9%) children were preschool age (39.04 ± 12.55 months) and 247 (75.1%) were school age (105.92 ± 29.12 months). With regard to religious affiliation, 43 of the preschoolers were MC and 39 were COC. In the other age group (5-14 years), 66 and 181 were MC and COC, respectively.

The overall incidence of appendicitis was 8.2/10,000 children per year (95% confidence interval CI = 5.8-10.5). Stratified analysis according to the children's religious affiliation revealed annual incidences of 6.7/10,000 MC (95% CI = 3.4-9.9) and 9.2/10,000 COC (95% CI = 6.0-12.5; *p *= 0.181). Regarding the annual incidence of acute appendicitis in preschool- and school-aged children according to religious affiliation, it was found that the annual incidence of acute appendicitis among preschoolers was 6.1/10,000 MC and 3.8/10,000 COC, respectively (95% CI = 1.2-11.0 and 1.0-7.1, respectively; *p *= 0.314), whereas the incidence among school-age children was 6.4/10,000 MC and 12.7/10,000 COC, respectively (95% CI = 2.2-10.5 and 7.8-17.6, respectively; *p *= 0.048). Among the entire cohort, the annual incidence of acute appendicitis was significantly higher among school-aged (10.1/10,000; 95% CI = 6.8-13.5) than among preschoolers (5.2/10,000; 95% CI = 2.3-8.2; *p *= 0.030); this difference was even more pronounced for COC (*p *= 0.009), but did not exist for MC (*p *= 0.429; Table 1). Moreover, the percentage of preschool MC (39.4%, 43/109) was significantly higher than the percentage of preschool COC [17.7%, 39/220; *p *< 0.001, adjusted OR (aOR) = 3.02, 95% CI = 1.80-5.07].

**Table 1:** Annual incidence of acute appendicitis per 10.000 children in preschool and school age in relation to the religion

Religion	Total	Preschool age	School age	*p *value
Greek Muslims	6.7	6.1	6.4	0.429
Greek Christians	9.2	3.8	12.7	0.007

*p *value	0.181	0.314	0.048	

Preschool age; children ≤ 5 years-old, School age; children > 5 - 14 years-old,

The characteristics of the preschoolers and the study factors are listed in Table 2 according to their religious affiliations. More than half of them were boys (61.0%). Almost 20% had no inside toilet facilities (18.3%) or hot water (19.5%) at home, while 39.0% of the children were living in overcrowded conditions. Most of the children were living in rural areas (70.7%), and 87.8% of them were consuming green vegetables almost every day. Forty-three (52.4%) were MC (26 males; age 39.07 ± 12.66 months, range 18-60 months) and 39 (47.6%) were COC (24 males; age 39.00 ± 12.59 months, range 17-60 months). The lack of inside toilet facilities at home (*p *= 0.018), overcrowded living conditions (*p *< 0.001), and living in a rural area (*p *< 0.001) were significantly more frequent among MC; no statistically significant differences between MC and COC were found for gender (*p *= 0.921), the existence of hot water at home (*p *= 0.145), the family history of acute appendicitis as a preschooler (*p *= 0.599), or vegetable consumption (*p *= 0.609). Discriminant forward stepwise analysis indicated that overcrowded living conditions (Wilk's lambda = 0.788, *p *< 0.001) and living in a rural area (Wilk's lambda = 0.787, *p *< 0.001) were factors that discriminated between MC and COC. These variables correctly classified 73.2% (60 out of 82) of the patients with acute appendicitis.

**Table 2:** Risk factors for acute appendicitis in preschool age in relation to children's religion

		Religion		
			
	Total n = 82 (%)	Greek Muslims n = 43 (%)	Greek Christians n = 39 (%)	*p *value
Male	50 (61.0)	26 (60.5)	24 (61.5)	0.921
No toilet facilities at home	15 (18.3)	12 (27.9)	3 (7.7)	0.018
No hot water at home	16 (19.5)	11 (25.6)	5 (12.8)	0.145
Overcrowded families	32 (39.0)	26 (60.5)	6 (15.4)	< 0.001
Living in rural area	58 (70.7)	39 (90.7)	19 (48.7)	< 0.001
Positive family history for AA	34 (41.5)	19 (44.2)	15 (38.5)	0.599
Vegetable consumption ≥ 5 t/w	72 (87.8)	37 (86.0)	35 (89.7)	0.609

Amount of lymphoid tissue	Total, *n *= 60 (%)	Greek Muslims, *n *= 26 (%)	Greek Christians, *n *= 34 (%)	0.002
low	22 (36.7)	3 (11.5)	19 (55.9)	
high	38 (63.3)	23 (88.5)	15 (44.1)	

A.A., Acute appendicitis

preschool age; children ≤ 5 years-old, t/w; times per week

**Figure 1:**
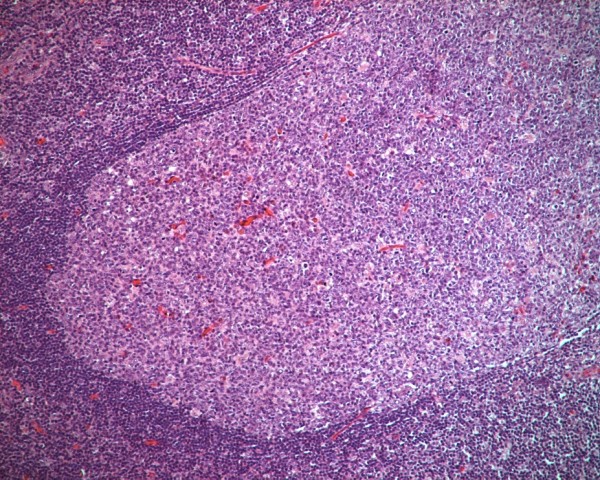
**An appendix with low amount of lymphoid tissue**. Histological assessment of the lymphoid tissue of the resected appendices.

**Figure 2:**
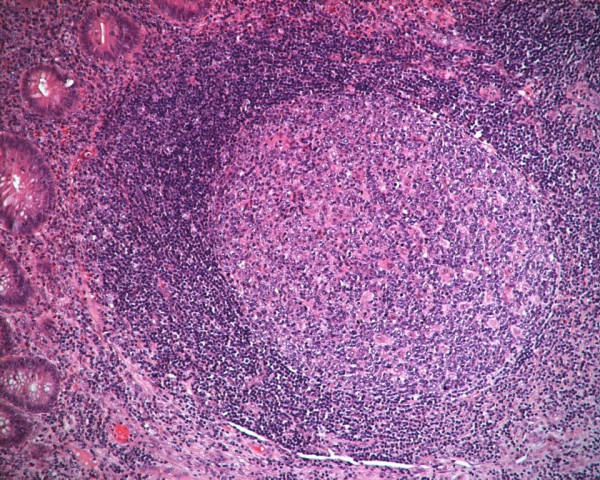
**An appendix with high amount of lymphoid tissue**. Histological assessment of the lymphoid tissue of the resected appendices.

Histological assessment of the lymphoid tissue of the resected appendices was feasible in 60 out of 82 specimens; total destruction of the histological structure of the appendiceal wall was observed in 22 (17 and 5 specimens from MC and COC, respectively). The total destruction of the histological structure of the appendiceal wall occurred significantly more frequently among the MC than among the COC (*p *= 0.006, aOR = 4.6, 95% CI = 1.5-13.6). Low and high amounts of lymphoid tissue were found in 22 (36.7%) and 38 (63.3%) of the specimens, respectively (Figure 1, Figure 2). The presence of a large amount of lymphoid tissue was significantly more common in children with no inside toilet facilities (*p *= 0.001) or hot water (*p *= 0.001) at home, those living in overcrowded conditions (*p *= 0.001) or in rural areas (*p *= 0.010), and in MC (*p *< 0.001). An elevated prevalence of large amounts of lymphoid tissue was also found among female patients, children with a positive family history of acute appendicitis, and children with infrequent vegetable consumption, but none of these differences were statistically significant (*p *= 0.185, *p *= 0.224, and *p *= 0.419, respectively). Multivariate logistic regression analysis revealed that the lack of inside toilet facilities at home (*p *= 0.047) and being of the Muslim faith (*p *= 0.029, aOR = 5.2, 95% CI = 1.2-23.2) remained independent risk factors for a large amount of lymphoid tissue (Table 3). In addition, the amount of lymphoid tissue in the appendix of preschool children who underwent incidental appendectomy was low and high in 3 and 17 MC, respectively, and in 11 and 9 COC, respectively; a large amount of lymphoid tissue was statistically significantly more common in MC (*p *< 0.05).

**Table 3:** The presence of high amount of lymphoid tissue in relation to children's characteristics

	High amount of lymphoid tissue	OR (95% CI)	*p *value
Gender			0.185
Male	23 (57. 5)	ref.	
Female	15 (75.0)	2.2 (0.7 - 7.3)	
Toilet facilities at home			0.001
Yes	23 (51.1)	n.a.	
No	15 (100.0)		
Hot water at home			0.001
Yes	24 (52.2)	n.a.	
No	14 (100.0)		
Overcrowded families			0.001
No	18 (47.4)	ref.	
Yes	20 (90.0)	11.1 (2.3 - 54.3)	
Place of living			0.010
Urban	7 (38.9)	ref.	
Rural	31 (73.8)	4.4 (1.4 - 14.3)	
Positive family history for AA			0.224
No	18 (56.3)	ref.	
Yes	20 (71.4)	1.9 (0.7 - 5.7)	
Vegetable consumption			0.419
< 5 times/week	4 (80.0)	2.5 (0.3 - 23.6)	
≥ 5 times/week	34 (61.8)	ref.	
Religious/ethnic group			< 0.001
Greek Christians	15 (44.1)	ref.	
Greek Muslims	23 (88.5)	9.7 (2.4 - 38.6)	

A.A., Acute appendicitis

Ref., reference category; n.a., not available

The mean durations of symptoms for MC and COC were 53 hours (range, 18-78 hours) and 49 hours (range,15-75 hours), respectively (*p *= 0.865). Among the entire cohort, the rate of complicated appendicitis (gangrenous or ruptured), as appraised by the pediatric surgeon's intraoperative assessment, was 69.5% (57 patients); the complication rate was 74.4% (32/43) among the preschool MC and 64.1% (25/39) among preschool COC (*p *= 0.311, aOR = 1.6, 95% CI = 0.6-4.2).

## Discussion

There is a general consensus that during the second half of the 20^th ^century, the worldwide incidence of acute appendicitis declined due to year-by-year socioeconomic improvements [13,14]. When this reduction in the incidence of appendectomy is considered according to age, it can be seen that the incidence fell from 3.6/10,000 to 1.1/10,000 among preschoolers, from 18.6/10,000 to 6.8/10,000 in children aged 5-9 years, and from 29.2/10,000 to 19.3/10,000 in children aged 10-14 years [15]. The "hygiene hypothesis" proposes that the decline of the incidence of acute appendicitis in the recent years is due to a reduced exposure to exogenous infections as a result of continued improvements in household amenities [16,11].

The overall annual appendicitis rate during the study period was 8.2/10,000 children, which is in accordance with the previously reported incidence [2]. As expected, the annual incidence of acute appendicitis was significantly higher in school-aged than in preschool children (*p *< 0.05), and only 82 cases occurred in the Thrace area in 7 years in a population of 57,614 children at risk, constituting an annual incidence of 5.2/10,000 children. This incidence agrees with Wilson et al. [17] reporting 81 cases of acute appendicitis in Belfast, UK, during a 7-year period among a population of 44,294 preschool-aged children.

In preschoolers, the reported percentage of acute appendicitis ranged from 4.5% to 15.6% of the total number of cases in children. Williams et al. [18] reported that they operated on 816 children for acute appendicitis during a 10-year period, of which 36 (4.5%) were < 5 years of age. Surana et al. [19] reported an analysis of the pathological findings of 954 patients with acute appendicitis, among which 63 (6.6%) were preschool children. Uba et al. [20] reported that 302 children underwent an appendectomy for acute appendicitis during a 10-year period, of which 44 (14.6%) were < 5 years of age. Alvarez et al. [21] reported that 288 children were operated on for acute appendicitis over a 17-month period, of which 45 (15.6%) were aged ≤ 5 years. In our study, although there was a trend toward a higher incidence of acute appendicitis among preschool MC than among COC, the difference did not reach statistical significance (*p *> 0.05). Nevertheless, in our study, acute appendicitis occurred three times more frequently among preschool MC than among preschool COC.

Several investigators have documented a higher incidence of acute appendicitis in preadolescents/adolescents and young adults [2]. In this age group, a proliferation of submucosal lymph tissue was observed in the appendix. An increase in the amount of lymphoid tissue in the appendiceal wall is thought to be the key determinant of local immunological and inflammatory responses to infectious or environmental agents, resulting in acute appendicitis. Our results indicate that in preschoolers who underwent appendectomy for acute appendicitis, the amount of lymphoid tissue in the appendiceal wall was significantly higher in MC than in COC (*p *< 0.05). It is unclear whether this difference is genetically determined in preschool MC or is the result of a more intensive immunological and inflammatory response or both; that is, the causal relationship is unknown. Nevertheless, the amount of lymphoid tissue in the removed appendices was significantly greater in preschool MC than in preschool COC who underwent incidental appendectomy (*p *< 0.05). Moreover, our results showed that the inflammatory process of the appendix was more vigorous in MC than in COC. Although the rate of complicated appendicitis was not significantly higher in MC than in COC (*p *> 0.05), the percentage of total destruction of the histological structure of the appendiceal wall was significantly higher in MC than in COC (*p *< 0.05). These phenomena may be due to disagreement between the pediatric surgeon's intraoperative classification of appendicitis and the pathologist's report [22].

The prevalence rates of inside toilet facilities, living in a rural area, and overcrowded conditions differed significantly between MC and COC (*p *< 0.05). The deprived hygiene conditions among MC in this age group probably rendered them vulnerable to infections, as improvements in hygiene have led to reduced rates of infection among young children [16]. This is supported by the increased percentage of admissions to the pediatric department of our hospital of preschool MC with respiratory and enteric infections compared to preschool COC suffering with the same infections. Moreover, a seasonal variation in the presentation of acute appendicitis in childhood has been noted, and this may be due to seasonal outbreaks of enteric infections [2]. A possible explanation as an immediate consequence of our results is that children aged ≤ 5 years who harbor a greater amount of lymphoid tissue in the appendiceal wall and live in conditions with a low standard of hygiene tend to develop acute appendicitis as preschoolers. These children are exposed to a higher rate of enteric infections that cause an immunologic reaction of the increased amount of lymphatic tissue of the appendix, leading to lumen obstruction and culminating in acute appendicitis. Exploration of this possible mechanism will require more detailed genetic and pathological studies.

The dietary habits of children are considered to be predisposing factors for the development of acute appendicitis. The "diet hypothesis" proposes that the style of diet (e.g., low in fiber and high in refined carbohydrate) is in some way responsible, since a high-fiber diet reduces stool transit times, reduces fecal viscosity, and inhibits fecalith formation. Several investigators have proposed that the fiber content of the diet or the effect of diet on the intestinal flora could be an important factor in the pathogenesis of appendicitis [23,24], but most studies on this issue have failed to find an association [25]. In our study, no statistically significant difference was found between the vegetable consumption of MC and COC; most of the children were reported by their mothers to consume vegetables every day. However, Brender et al. [26] reported that a high fiber intake was negatively associated with appendicitis among children > 6 years old but not among children aged less than 6 years.

A child is more prone to experience acute appendicitis if one or more close relatives have required an appendectomy [8]. A history of appendicitis in a first-degree relative is associated with a 3.5-10.0 relative risk for developing acute appendicitis. The strongest familial associations have been noted when children develop appendicitis at age < 6 years [1]. In our study, this factor did not differ significantly between preschool MC and COC (*p *> 0.05).

## Conclusions

In summary, although the retrospective analysis of our epidemiological data does not make it possible to draw an unequivocal conclusion regarding the clarification of the higher prevalence of acute appendicitis among preschool MC than COC, our results support that hypothesis that the development of acute appendicitis as a preschooler is influenced primarily by the amount of lymphoid tissue in the appendiceal wall and the hygiene conditions at home, rather than by the vegetable consumption or the family history of acute appendicitis. An environment with poor hygiene renders small children vulnerable to respiratory and enteric infections that, in addition to the increased amount of lymphoid tissue in the appendiceal wall and probably vigorous immunologic reactivity, may be responsible for the higher prevalence of acute appendicitis among preschool MC.

## Consent

Written informed consent was obtained from the patients' parent for publication of this paper and accompanying images.

## List of abbreviations

MC: Muslim children; COC: Christian Orthodox children

## Competing interests

The authors declare that they have no competing interests.

## Authors' contributions

**SG**: conceived and designed the study, analyzed the data, and drafted the final report; **AA**: participated in the design of the study, made the histological evaluations and contributed to the writing of the report; **KK**: participated in the design of the study, collected the data and has made useful contribution in drafting the manuscript and in the revision of the literature; **GT**: made statistical analysis, interpretation of epidemiological data and contributed to the writing of the report; **ES**: contributed to the writing of the report and revised the paper critically for important intellectual content; **GV**: helped with study design, contributed to the writing of the report and made the final approval of the version to be published.

All authors confirm that have read and approved the final manuscript.
